# Advances in Meta-Optics and Metasurfaces: Fundamentals and Applications

**DOI:** 10.3390/nano13071235

**Published:** 2023-03-30

**Authors:** Kai Ou, Hengyi Wan, Guangfeng Wang, Jingyuan Zhu, Siyu Dong, Tao He, Hui Yang, Zeyong Wei, Zhanshan Wang, Xinbin Cheng

**Affiliations:** 1 Institute of Precision Optical Engineering, School of Physics Science and Engineering, Tongji University, Shanghai 200092, China; 2 MOE Key Laboratory of Advanced Micro-Structured Materials, Shanghai 200092, China; 3 Shanghai Frontiers Science Center of Digital Optics, Shanghai 200092, China; 4 National Research Center for High-Efficiency Grinding, College of Mechanical and Vehicle Engineering, Hunan University, Changsha 410082, China; 5 Shanghai Institute of Intelligent Science and Technology, Tongji University, Shanghai 200092, China

**Keywords:** meta-optics, light manipulation, metalens, computational imaging, image processing

## Abstract

Meta-optics based on metasurfaces that interact strongly with light has been an active area of research in recent years. The development of meta-optics has always been driven by human’s pursuits of the ultimate miniaturization of optical elements, on-demand design and control of light beams, and processing hidden modalities of light. Underpinned by meta-optical physics, meta-optical devices have produced potentially disruptive applications in light manipulation and ultra-light optics. Among them, optical metalens are most fundamental and prominent meta-devices, owing to their powerful abilities in advanced imaging and image processing, and their novel functionalities in light manipulation. This review focuses on recent advances in the fundamentals and applications of the field defined by excavating new optical physics and breaking the limitations of light manipulation. In addition, we have deeply explored the metalenses and metalens-based devices with novel functionalities, and their applications in computational imaging and image processing. We also provide an outlook on this active field in the end.

## 1. Introduction

Light is the basis of human exploration and understanding of the world, because of carrying an enormous amount of information that can be perceived by people or machines. Flexibly and effectively controlling light beams has always been a significant goal that people aspire to achieve. According to the Huygens–Fresnel principle, the key to controlling light beams is to effectively design and modulate electromagnetic wavefronts [1,2]. Through manipulating the intrinsic dimensions of light waves (phase, amplitude, and polarization), information and energy can be effectively transferred and transformed in the light–matter interaction. Conventional optical technologies implement wavefront shaping based on phase accumulation from the propagation of light in a medium. The capacities of wavefront control are commonly limited by the conventional laws of refraction/reflection and the fabrication materials, resulting in optical components being too bulky to allow advanced functionalities in on-chip optical and photonic systems [3,4,5,6]. Metamaterial technologies based on subwavelength optics [7,8,9,10,11,12,13,14,15,16,17] demonstrate powerful abilities to control electromagnetic waves beyond the limitations of conventional optical technologies, and produce unconventional physical phenomena such as negative refraction [18,19,20,21,22,23,24,25,26,27], inverse Cherenkov radiation [28,29,30,31,32], and sub-diffraction limits [33,34,35,36,37,38,39]. The Science journal listed metamaterial technologies as one of the top 10 scientific and technological breakthroughs in the first decade of this century in 2010 [40].

Recently, meta-optics based on two-dimensional metamaterials (subwavelength-patterned surfaces, called metasurfaces) provide extreme miniaturization of optical components with multi-functionalities [41,42,43,44,45,46,47,48,49,50,51,52,53,54]. Metasurfaces have the significant advantage of simple processing compared to three-dimensional metamaterials and are promising for integration with on-chip nanophotonic devices benefiting from their planar and light configurations. Furthermore, optical metasurfaces have attracted extensive attention due to their powerful and on-demand control over the phase, amplitude and polarization of light beams [55,56,57,58,59,60,61,62,63,64,65,66]. The optical functionalities of metasurfaces can be flexibly and elaborately engineered through the tailoring the scattering behaviors of subwavelength resonators (meta-atoms) rather than refraction. Previous efforts often focused on metallic metasurfaces [67,68,69,70,71,72,73,74,75,76,77], which can enhance light–matter interactions and introduce versatile control of incoming light by activating local plasmonic resonance, albeit with limited efficiency due to ohmic loss [78,79,80,81,82,83]. Dielectric metasurfaces overcome the limitation of metallic metasurfaces, which have provided a promising way to develop highly efficient and multi-functional meta-devices [84,85,86,87,88,89,90,91,92,93,94,95,96,97,98,99,100].

Recent advances in meta-optics have been applied in the demonstrations of many exotic optical manipulations and various useful meta-devices [101,102,103,104,105,106]. Metasurface-based applications are promising alternatives to replacing existing optical devices, owning to the ultrathin, ultracompact, and multifunctional properties of meta-devices based on the principles of meta-optics [107,108,109,110,111,112,113,114,115,116]. Meta-optics offer the possibility to address the limitations of conventional optics and a new paradigm to achieve on-chip integrated meta-devices with multi-functional and parallel control of light beams [117,118,119,120,121,122,123,124,125]. Optical metalenses are predicted to have the potential to produce disruptive applications due to the integration of advanced imaging, and the miniaturization and multi-dimensional manipulation of meta-optics [126,127,128,129,130]. Metalens-based imaging technology was listed again in science’s top 10 scientific advances in 2016 [131]. Furthermore, multi-functional metalenses (such as the spiral metalens) with structuring point spread functions (PSF) can simultaneously implement edge-enhanced imaging and control of phase and polarization [41,44,52,132,133,134,135,136,137]. Existing efforts have pushed meta-optics into a new era from initial optical control and optical imaging to optical computing via performing metasurface-based mathematical operations on the optical field (including its amplitude, phase, polarization, and frequency, etc.) [85,124,138,139,140,141,142].

**Figure 1:**
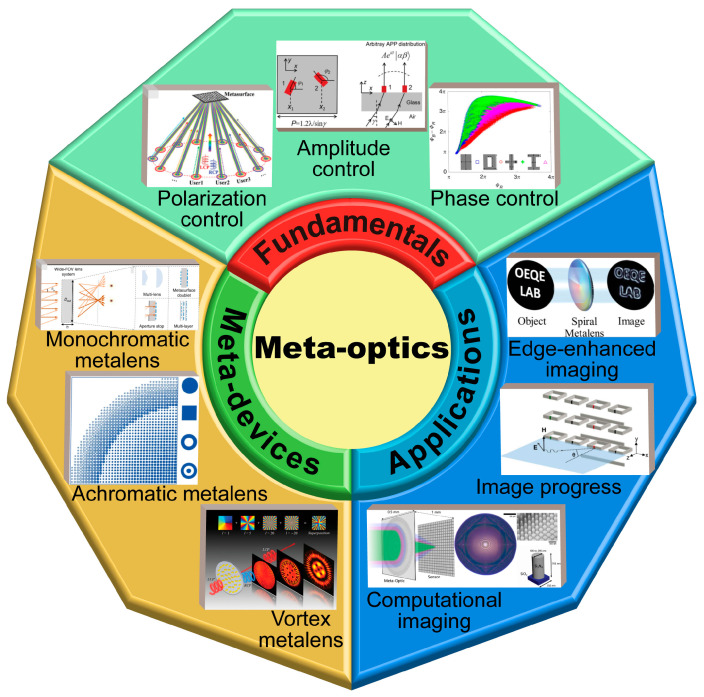
Fundamentals of meta-optics, meta-devices and applications. Overview of the recent development of meta-optics, including the control principle, meta-devices, and further meta-imaging [100,147,148,149,150,151,152,153,154].

With the deepening of research of meta-optical physics and vector light field regulation, the field of meta-optics has gradually shifted from one of basic research to one of practical application [143,144,145,146,147]. One of the main directions of meta-optics stands a good chance of exploring the limit of light field control, integrating multi-functional computational imaging and parallel image progressing in on-chip systems, and improving the working efficiency and bandwidth of optical meta-devices within a few years. As shown in Figure 1, the review aims to map the active research areas in the field over the past ten years, identify the key principles of meta-optics, and discuss advanced meta-devices and their latest applications in advanced imaging and image processing.

## 2. Fundamentals

### 2.1. Phase Control

**Figure 2:**
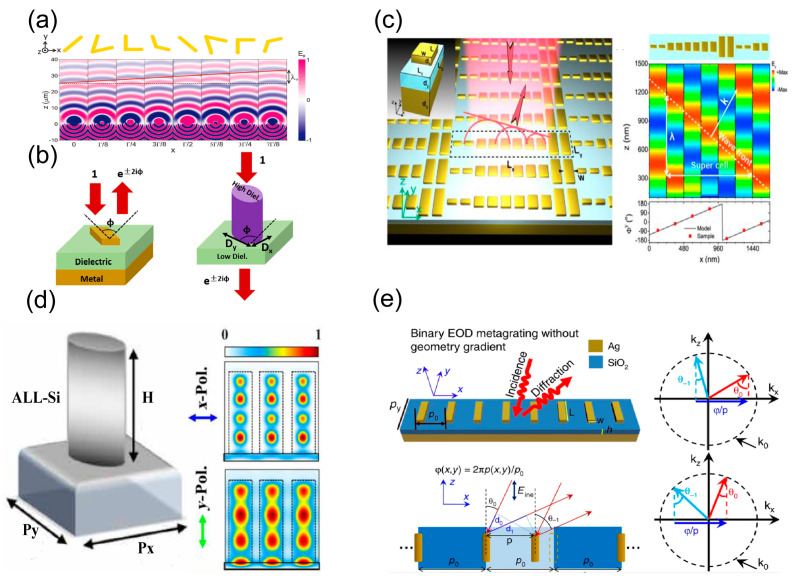
Phase control for metasurfaces. (**a**) V-shaped antenna array for demonstration of generalized laws of reflection and refraction [51]. (**b**) Geometric-phase-based metasurface [155]. (**c**) Resonant-phase-based metasurface for wave-shaping [156]. (**d**) The principle of the propagation phase based on monocrystalline Si meta-atoms [157]. (**e**) Schematics used to illustrate the principle of the detour phase that is proportional to the displacement, p [158].

Optical metasurfaces can efficiently and flexibly manipulate the wavefront of light beams by spatially tailoring the optical response of meta-atoms (phase, amplitude and polarization, etc.) at a subwavelength scale. Due to the suppression of high-order diffraction, the control efficiency can be close-to-unity. Prof. Capasso et al. proposed the relevant definition of the metasurface for the first time in 2011. As shown in Figure 2a, by introducing the phase discontinuities via V-shaped nano-antennas, the generalized laws of reflection and refraction have been demonstrated [51]. Figure 2b demonstrates the common geometrical configurations of reflective and transmissive meta-atoms (symmetrical strips and elliptical nano-pillars). Considering a nano-structure with a rotation angle (ϕ) under normal incidence, the reflected or transmitted light beams can obtain a phase delay of ±2ϕ relative to the incidence. Here, let us discuss the fundamental principle of phase control with the transmissive metasurface as an example. The optical response of the anisotropic meta-atoms can be expressed as follows [155]:

(1) $$J=R(-\phi )\left(\begin{array}{cc} A_{mx}e^{i\phi^{mx}} & 0\\ 0 & A_{my}e^{i\phi^{my}} \end{array}\right)R(\phi )$$

when the incident light filed is the circularly polarized (CP), wave ${\hat{e}}_{\pm }=\frac{1}{\sqrt{2}}\left({\hat{e}}_{x}\pm i{\hat{e}}_{y}\right)$, and the transmitted wave $E_{t}^{\pm }=J{\hat{e}}_{\pm }$. Following Equation (1), we can obtain the final transmitted light filed:

(2) $$E_{t}^{\pm }=\frac{1}{2}\left(A_{mx}e^{i\phi^{mx}}+A_{my}e^{i\phi^{my}}\right){\hat{e}}_{\pm }+\frac{1}{2}\left(A_{mx}e^{i\phi^{mx}}-A_{my}e^{i\phi^{my}}\right)e^{\pm i2\phi }{\hat{e}}_{\mp }$$

where $R(\phi )=\left(\begin{array}{cc} cos\phi & sin\phi \\ -sin\phi & cos\phi \end{array}\right)$ is the two-dimensional rotation matrix, $A_{mx}e^{i\phi^{mx}}$ and $A_{my}e^{i\phi^{my}}$ are the complex transmitted amplitudes of the meta-atoms for x- and y-polarization incidence, respectively. Following Equation (2), we can find that transmitted light includes two kinds of circularly polarized states. The handedness of the first term is the same as the incident CP beam without any phase delay, which is called a co-polarized component. The second one is a cross-polarized component with a phase delay of ±2ϕ, but the handedness is reversed. By selecting appropriate structural parameters, we can always make $A_{mx}\approx A_{my}=A_{0}$. Defining $\delta =\phi^{mx}-\phi^{my}$, $\phi^{0}=\phi^{mx}-\phi^{my}$, we can obtain the following equation:

(3) $$E_{t}^{\pm }=\frac{A_{0}e^{i\phi_{0}}}{2}\cos\frac{\delta }{2}{\hat{e}}_{\pm }+i\frac{A_{0}e^{i\phi_{0}}}{2}\sin\frac{\delta }{2}e^{\pm i2\phi }{\hat{e}}_{\mp }$$

From Equation (3), the ratio of the co-polarized and cross-polarized components can be regulated by adjusting the polarization-dependent transmission phases $\phi^{mx}$ and $\phi^{my}$. When δ=±π (i.e., when the meta-atoms can be half-wave plates), the incidence is completely converted into the cross-polarized components (the conversion efficiency is 100%), and a ±2ϕ phase shift (i.e., geometric phase) can be obtained. Based on the geometric phase, full 2π phase control can be realized via rotating the orientation angle (ϕ) from 0 to π.

The resonant behavior and near-filed modes of the meta-atom can be effectively controlled through changing the geometric structures of the meta-atoms. As shown in Figure 2c, wave-front shaping with full 2π phase control can be realized with meta-atoms of different geometric structures [156]. In Figure 2d, the transmission phase (or dynamic phase) is manipulated by elaborately engineering the waveguide mode of the dielectric nano-pillars, which is proportional to the height of the nano-pillars [157]. These metasurfaces are called high-refractive-index contrast metasurfaces, and commonly fabricate high-refractive-index dielectric (such as Si, GaN, etc.) patterns on the dielectric substrate with a low refractive index (SiO2, Al2O3, etc.) [158]. Through changing the relative position of the meta-atoms and elaborately regulating the plasmonic resonance mode, the detour phase can be capable of achieving highly efficient diffraction in a specific diffraction order, as shown in Figure 2e [158].

### 2.2. Amplitude Control

**Figure 3:**
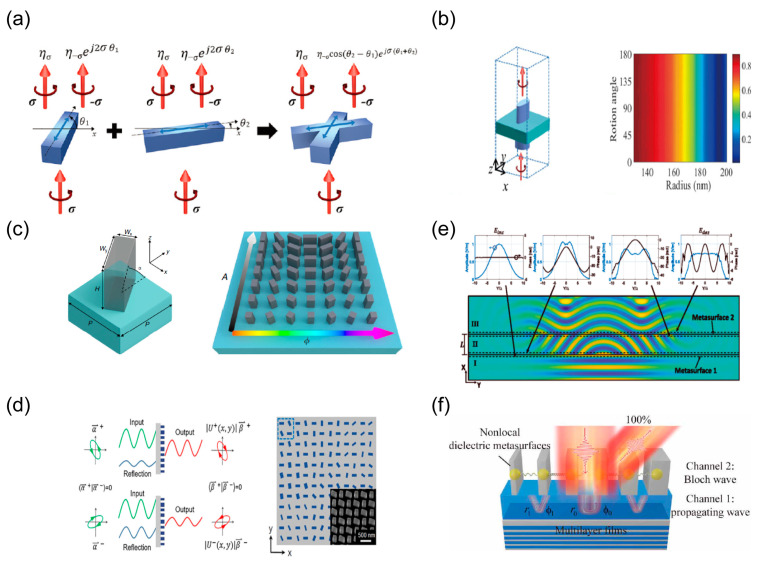
Amplitude control of metasurfaces. (**a**) Schematic of regulation mechanism with X-shaped meta-atoms [160]. (**b**) Amplitude control enhanced by using Fabry–Perot resonance of the sandwich configuration [161]. (**c**) Decoupling the amplitude and phase by controlling polarization conversion efficiency and geometrical degree of freedom of the birefringent meta-atoms [100]. (**d**) Interference meta-molecules used to impose two independent amplitude profiles on any pair of orthogonal states of polarization [162]. (**e**) Principle of double layers of non-local metasurfaces manipulating amplitude without loss [163]. (**f**) Schematic of all-dielectric quasi-three-dimensional non-local meta-grating [142].

Arguably, existing efforts in meta-optical physics are often centered on manipulating the phase that is considered the soul for the design of the wavefront. However, simultaneously encoding the amplitude and phase is needed for high-performance meta-devices (such as holography, structured light manipulation, etc.). Expanding the gamut of achievable flat optical devices requires control of more than just the phase. For this reason, recent efforts have pushed the limitations of the simultaneous control of more than one parameter at a time. Therefore, amplitude control originating from the redistribution of incident power is of significant importance to achieving highly efficient and multi-functional meta-devices. Recently, most design strategies have generally achieved amplitude control using the reflective loss, the polarization loss and the coherent loss based on the phase control [159]. From Equation (3), anisotropic meta-atoms carrying geometrical phase can achieve full control of the amplitude of the cross-polarized component by freely regulating the polarization conversion efficiency. As shown in Figure 3a, Byoungho Lee et al. propose X-shaped meta-atoms which can be regarded as the superposition of two nano-rods with different orientation angles $\left(\theta_{1},\theta_{2}\right)$. The transmitted amplitude of the cross-polarized component is proportional to $2\cos(\theta_{1}-\theta_{2})$, resulting in full amplitude control by adjusting the angular disparities of X-shaped meta-atoms [160]. However, the modulation efficiency is less than 50% due to the cross-talk among X-shaped meta-atoms. In Figure 3b, amplitude control enhanced by using Fabry–Perot resonance of the sandwich configuration is demonstrated [161]. By altering the radius of the nano-cylinders to change the F–P resonance, the polarization conversion efficiency and purity of the sandwich structure are finally close to 90%. As shown in Figure 3c, by increasing geometrical degrees of freedom and elaborately manipulating the polarization efficiencies of the dielectric birefringent meta-atoms, Nanfang Yu et al. proposed a general scheme of complex amplitude control with high efficiency [100]. They implemented 3D monochromatic complex amplitude holograms of high quality using fully dielectric metasurfaces.

The above amplitude control methods are limited to CP incidence and require complex geometrical configurations that come with computational costs. However, due to the inherent symmetry of the geometrical phase for birefringent meta-atoms, amplitude response cannot be distinguished from two spins. Amplitude control is essentially a Hermitian modulation on an incoming wave. As shown in Figure 3d, Ting Xu et al. illustrate the principle of decoupling amplitude and polarization for any pair of orthogonal polarizations. The Hermitian matrix for simultaneously controlling polarization and amplitude can be obtained by combining several nano-pillars into a meta-molecule based on coherent loss and singular value decomposition (SVD) [162]. They propose the Hermitian Jones matrix associated with orthogonal polarization states and independent amplitude profiles from the perspective of interference by combining the geometric phase and propagation phase. The prosed methodology to obtain the desired field pattern based on loss control comes at the expense of the total transmitted power. As shown in Figure 3e, researchers developed non-local meta-optical physics using the combination of two closely spaced reflectionless metasurfaces (compound metasurfaces) which avoid the loss of reflection, absorption and polarization conversion. Reflectionless-compound meta-optics first reshapes the incident power flow into the desired profile and then provides phase correction. It successfully implements amplitude reshaping for arbitrary wavefronts [163]. By elaborately modulating the non-local optical response from inter-layer coupling and the in-plane, Cheng et al. developed all-dielectric quasi-three-dimensional meta-grating with perfect anomalous reflection, as shown in Figure 3f. They demonstrated the highest anomalous reflection efficiency in both the design and experiment to date. Recent advances have experimentally demonstrated that complex amplitude can implement high-performance 3D holograms and control the generation of structured light beams [164,165]. Moreover, digitally encoded metasurfaces can simplify the design of metasurfaces, and digital and active metasurfaces enable the integration of tunable metamaterial technologies with digital signals and image processing [166].

### 2.3. Polarization Control

**Figure 4:**
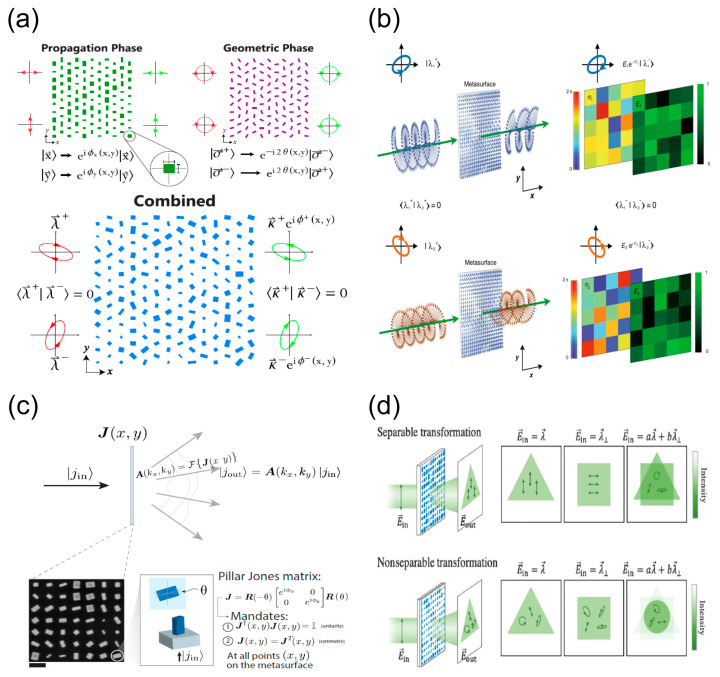
Polarization control using form-birefringent metasurfaces. (**a**) Control of orthogonal polarizations by combining the propagation and geometric phases [167]. (**b**) Complete and independent control of the complex amplitude for orthogonal polarization states [168]. (**c**) Jones matrix holography using birefringent metasurfaces [169]. (**d**) Principles of non-separable and separable polarization wavefront transformations [170].

Metasurfaces with multi-functional wavefront control show the significant superiority of meta-optics [109]. Generally, anisotropic and sub-wavelength meta-atoms act as waveplates due to their vastly different responses to orthogonal polarizations [55]. Metasurfaces, composed of anisotropic meta-atoms, demonstrate the properties of polarization-dependent control. Following Equation (3), we can simultaneously and independently control the wavefront phase profiles for arbitrary orthogonal polarization states by combining the propagation phase and the geometric phase of the birefringent meta-atoms. As shown in Figure 4a, Capasso et al. illustrate the decoupling mechanism between the polarization and phase [167]. By elaborately designing the spatial arrangement of the compound meta-atoms and introducing a polarization-dependent interference mechanism, Tingxu et al. propose a general scheme for simultaneously and independently manipulating the complex amplitudes for polarization pairs of orthogonal states [168], as shown in Figure 4b. Based on the strategy of combining the Jones matrix’s phase retrieval and the matrix polar decomposition, the Jones matrix holography metasurface has been demonstrated in Figure 4c. The demonstrated holograms implement parallel polarization analysis and custom waveplate-like behavior with the method of multi-channel wavefront control [119,169]. On the basis of the matrix’s meta-optics [114], Figure 4d demonstrates non-separable polarization wavefront transformations using double-layer form-birefringent metasurfaces, which overcomes the intrinsic limitations of single-layer ones [170].

## 3. Meta-Devices

### 3.1. Mononchromatic Metalens

**Figure 5:**
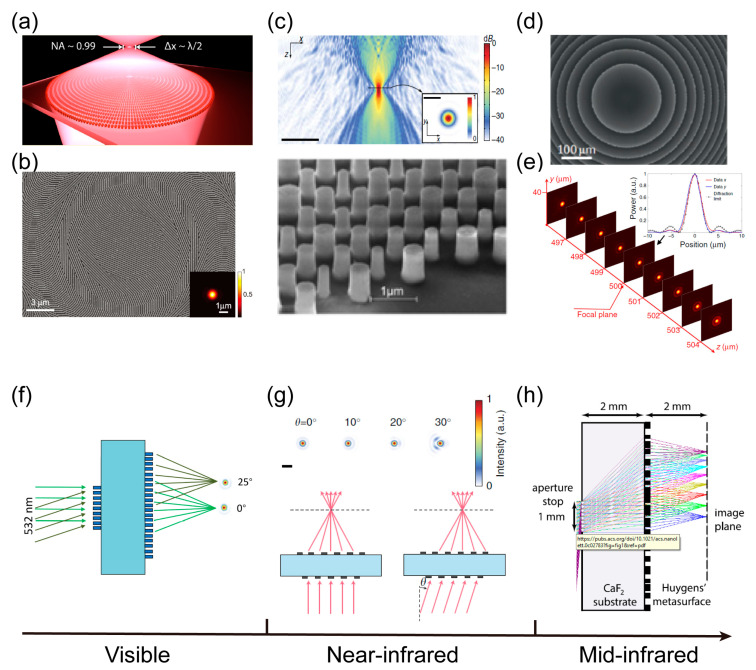
Metalenses used for monochromatic aberration correction. (**a**) Metalens with high numerical aperture (NA ≈ 1) [172]. (**b**) Geometric phase-based metalens with high focusing efficiency [47]. (**c**) Near-infrared polarization-insensitive metalens [173]. (**d**) Polarization-insensitive metalens with high contrast metasurface [177]. (**e**) Ultra-thin metalens via Huygens metasurface, and the focal spot profile evolution along the optical axis at 5200 nm wavelength [174]. (**f**–**h**) Wide field-of-view monochromatic aberration-corrected metalenses in the (**f**) visible wavelength [175], (**g**) near-infrared [176], and (**h**) mid-wavelength infrared [129].

Optical lenses play a key role in modern optoelectronic applications. Among them, lenses with large numerical aperture (NA), wide field of view, and high focusing efficiency determine the performance of optical systems in some import applications, such as lidar, microscope objectives, and photography camera. In the design of conventional refractive or diffractive optics, the configurations cascading multiple lenses are commonly used to correct the chromatic aberration of the system. Therefore, the conventional optical systems are too bulky and difficult to adapt to the miniaturization of optical systems and also require high manufacturing costs. For instance, commercial company Leica have developed an alignment equipment weighing about six tons only to align a microscope objective system of about one kilogram [171]. Optical metalenses underpinned the meta-optics provides a promising platform to overcome the limitations in their conventional counterparts. Diffraction-limited imaging is achieved using a single-layer metalens, with large NA metalenses applied to small light-matter interaction volumes or large angular collections. As shown in Figure 5a, metalens with a large numerical aperture (NA > 0.99) and subwavelength thickness (λ/3) in transmission mode has been achieved by elaborately engineering resonant scattering effects of asymmetric meta-atoms in the visible. Focusing efficiency is defined by the ratio of the power at the focal point to the power of the incident light. The diffraction angles of light near the edge of the metalenses increase with increasing NA, requiring phase shifts in deep-wavelength spatial revolutions. The phase errors due to coupling between adjacent elements are unavoidable. These two factors lead to a reduction in the efficiency at the large deflective angles, which leads to a reduction in the focusing efficiency. Using the design of geometric phase-based metagrating [172], circular-polarization-sensitive metalens with large NA and high efficiency [47] has been realized, shown in Figure 5b. In general, the focusing efficiency of the metalenses decrease as the increase of NA due to the enhanced coupling among the meta-atoms. Figure 5c,d demonstrate polarization-insensitive metalenses with a high efficiencies (~80%) and large NAs (~0.8) [173] via the angle-insensitivities of symmetry dielectric nanopillars. Based on the design of Huygens metasurface (resonate phase control mentioned in Figure 2), Hu et al. develop mid-wavelength infrared metalens with ultra-thin thickness (λ/8) and high focusing efficiency via chalcogenide alloy PbTe metasurfce platform [174]. The ultra-thin metalens demonstrates high-quality imaging comparable to that of commercial lenses. Wide field-of-view imaging is required in imaging applications such as AR, microscopy, landscape imaging, and image projection. Limited field of view due to unavoidable aberrations at oblique incidence with concomitant loss of focusing efficiency. Figure 5f–h demonstrate metalenses correcting the monochromatic aberrations in the visible band [175], near-infrared band [129,176], and mid-wave infrared band [129], respectively. The wide field-of-view metalenses have been designed by combination of meta-optical wavefront control and the ray tracing approach, and high-quality imaging has been achieved under wide-angle range incident irradiation.

### 3.2. Broadband Achromatic Metalens

**Figure 6:**
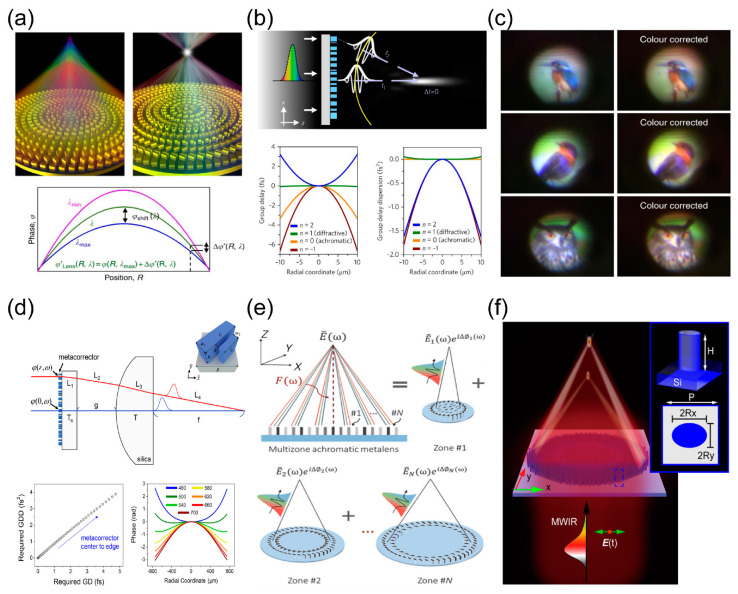
Broadband achromatic metalenses. (**a**) Schematics of comparison between chromatic (left) and achromatic (right) metalenses [50]. (**b**) Principle of broadband achromatic metalenses [181]. (**c**) Fullcolor imaging with (right) and without (left) an achromatic metalens [182]. (**d**) Schematic of hybrid metalens consisting of a meta-corrector with dispersion control and a commercial spherical lens [183]. (**e**) Principle of RGB-achromatic metalens with constructive interference and dispersion engineering [103]. (**f**) Schematic of varifocal achromatic metalens which can achromatically focus mid-wavelength infrared beams in different focal planes according to the incident polarization states [184].

The monochromatic metalenses perform greatly at a desired single wavelength; however, their broadband functionalities, such as focusing and full-color imaging, commonly suffer from the wavelength dispersion characteristics of meta-atoms which are not considered and engineered in the design of monochromatic metasurfaces. The dispersion results in separation of the focal spots of the metalens under broadband incidence; that is light beams with different wavelengths are focused into different positions along the optical axis, affecting its imaging performance (e.g., imaging blur). Physically, a broadband light beam can be understood as a wave-packet with carrier frequency ($\omega_{0}$) and a certain amount of broadening along the time axis. In order to make wave-packets that interact with meta-atoms at different metalens positions achieve the same focus at the same time, the total time delay (group delay) of the wave-packets modulated by the metalens must be constant (wavelength-independent), and the phase delays (carrier phase) from different meta-atoms at the carrier frequency must satisfy the coherence condition at the focal point, as shown the schematic in Figure 6b. Based on this broadband achromatic methodology, various novel achromatic meta-devices have been proposed by tailoring the dispersion and resonant behaviors of meta-atoms rather than the material dispersion [128,178,179,180].

As shown in Figure 6a, Din Ping Tsai et. al constructed a reflective broadband achromatic metalens by simultaneously and independently manipulating the carrier phase and the group delay phase via linear phase dispersion combined with the geometric phase and resonate phase [50]. Furthermore, Capasso et al. proposed a general achromatic design by engineering the phase profile, group delay, and group delay dispersion of meta-atoms, as shown in Figure 6b. They proposed a transmissive achromatic metalens with the large visible bandwidth of 470 to 670 nm. Figure 6c shows the full-color imaging of an achromatic metalens that was accomplished by introducing integral resonances to achieve the required phase compensation [182]. Limited by the broadband achromatic principle and micro-nano processing technology, achromatic metalenses suffer from the balance between sample size, NA, and working bandwidth. The actual resolution of conventional lenses is reduced due to the presence of aberrations. However, metalenses have local phase control ability within a sub-wavelength scale, compensating for the aberrations of traditional lenses. Figure 6d shows a design of a meta-corrector to correct spherical aberration and chromatic aberration in commercial spherical plano-convex lenses by elaborately tailoring the dispersion behaviors of the meta-atoms used in the meta-corrector [183]. Figure 6e demonstrates a large-area, multi-wavelength RGB-achromatic metalens of a millimeter-scale diameter [103], which has achieved a compact virtual reality (VR) platform and near-eye fiber-optic scanning. Furthermore, researchers developed a polarization-controlled varifocal metalens by decoupling polarization, phase, and dispersion, as shown in Figure 6f. Additionally, a polarization-insensitive broadband achromatic metalens with a large NA and sample size has been achieved in the mid-wavelength infrared region. They successfully implemented broadband achromatic imaging [184]. At present, there are still challenges in the research of achromatic metalenses such as large NAs and sample sizes, and multi-functionality. Large-aperture, dynamically tunable multi-functional broadband achromatic metalenses are expected to produce important applications in endoscopy and biological imaging, etc. As extremely narrow counterparts to broadband metasurfaces for controlling wavelength and spatial dispersion, BIC metasurfaces exhibit behaviors with high q-factor wavefront control and are abnormally sensitive to changes in the dielectric environment around them. The excellent selection and sensitivity to the spectrum can be applied to bio-sensing and refractive index sensors [185,186].

### 3.3. Multi-Functional Meta-Devices

**Figure 7:**
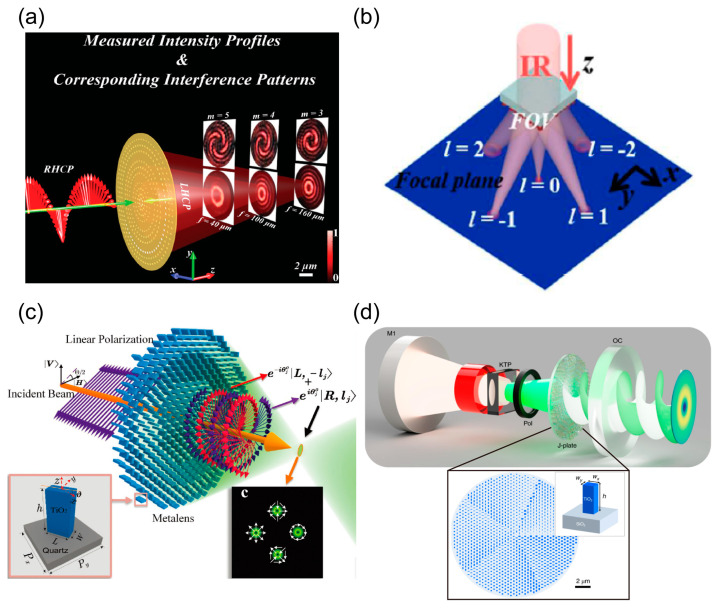
Generation and detection of optical vortices based on metasurfaces. (**a**) Generation of coaxial multi-channel optical vortices with metasurface [195]. (**b**) Schematic of the focusing optical vortex generator operating in transmission mode [196]. (**c**) Metasurface for generation of multi-channel vector optical vortices [197]. (**d**) Metasurface optical vortex laser [198].

The optical vortex (OV) is a structured light beam carrying orbital the angular momentum (OAM) of a photon. The light beam has attracted much attention since its discovery by L. Allen in the 1990s [187]. Such OAM-carrying structured light beams have been found to have various significant applications in optics and photonics, such as optical communications [188], optical trapping [189], and image processing [190], etc. Because of their properties of ultra-compact configuration and powerful functionalities for wavefront control, metasurface-based structured-light technologies offer new opportunities for excavating the fundamentals and applications of light beams that are impossible to achieved by conventional optics [191,192,193,194]. As shown in Figure 7a, Shuang Zhang et al. propose multichannel metalenses via geometrical phase-based metasurfaces composed of metallic nanovoid arrays. They successfully implement the generation of multi-channel focusing OV with hollow-shaped PSFs [195]. When the vortex light is incident with a different vorticity, the spin angular momentum and the position of the focal plane can be controlled. OAM beams with different topological charges can be observed in different focal planes. Therefore, it has potential application value in the precise sorting operation of nano-particles. Experimentally, the number of topological charges has been characterized by using the interference method. By researching the wavefront evolution form satisfied by the space-structured light field, the wavefront phase distribution function of the meta-interface is established, the meta-atoms with centrosymmetry are designed to reconstruct these phase profiles of the wavefront, and a plane wave of any polarization can be converted into a vortex beam by orbital angle momentum and focusing it onto a specific focal plane. In Figure 7b, Kai Ou et al. demonstrate a polarization-independent spiral metalens that can efficiently perform the detection and generation of multi-channel focusing OV beams in the near-infrared region [196]. Arbitrarily polarized plane waves can be converted into focusing optical vortex beams with high efficiencies of 70–85%. Multi-channel meta-devices have successfully implemented the detection of topological charges from −2 to 2. Figure 7c shows a polarization-dependent multi-channel metalens capable of implementing OV mode-multiplexing and demultiplexing [197]. The OAM state and polarization distributions can be simultaneously controlled in three different multiplexing types of lattices (such as triangular, square, and rhomboid lattices). As shown by Figure 7d, Capasso et al. developed a geometrical phase-based OV meta-device to obtain high-purity OAM laser beams based on the decoupling mechanism between the polarization and phase demonstrated in Figure 4a [198]. Due to its efficient and flexible optical field manipulation capability and subwavelength thickness of the above optical vortex meta-devices, it has paved a promising way to developing various compact integrated optical systems, such as biomedical chips, quantum key distribution, and so on.

## 4. Applications

### 4.1. Computational Imaging

**Figure 8:**
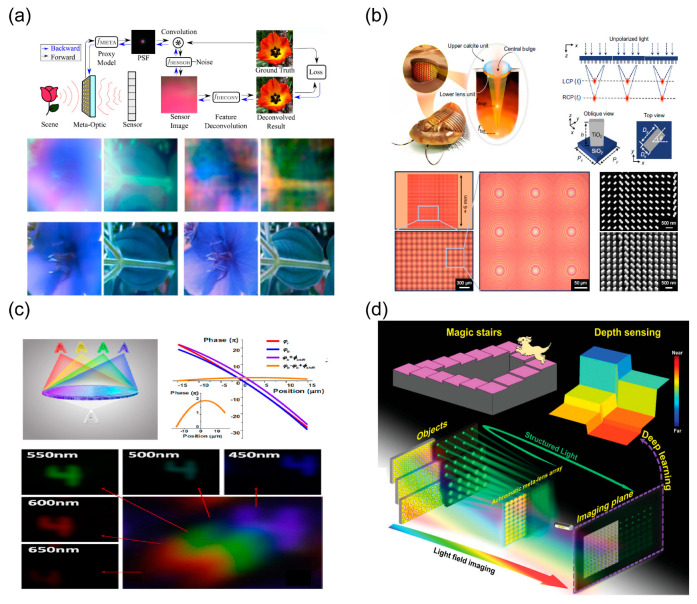
Metalens-based computational imaging. (**a**) Computational imaging using an optimized phase profile metalens and the deep learning reconstruction algorithm has enabled the reduction in monochromatic and chromatic aberrations [150]. (**b**) A nano-photonic light-field camera inspired by Trilobite that uses a spin-multiplexed bi-focal metalens array to deeply increase the depth of field [199]. (**c**) Spectral light-field imaging achieved by using a transversely dispersive metalens [200]. (**d**) Schematic of an achromatic metalens array’s depth-sensing system [201].

Benefiting from the properties of the implementation of the on-demand design of optical wavefronts using metalenses and their multi-functionality, metalens-based imaging enhanced by computational imaging and the learning framework has produced revolutionary applications in miniature optical systems. In the computational imaging framework, the intensity profiles captured by sensors are blurry and are typically not direct images of the object, and imaging information about the target objects is obtained by processing the captured intensity patterns. By modulating the PSF using metalenses with asymmetric phase profiles (such as cubic focusing) and performing deconvolution processing, researchers proposed a scheme to increase the bandwidth of imaging systems. Figure 8a demonstrates an end-to-end differentiable learning framework for the design of the meta-optical imager. Full-color and wide-FOV imaging can be achieved by optimizing symmetric PSF and performing a neural-network-based image reconstruction process [150]. As shown in Figure 8b, the polarization-controlled bifunctional array of metalenses is designed to achieve varifocal imaging used in light-field cameras. Based on the varifocal meta-optical system, the neural-network-based reconstruction method is used for engineering chromatic dispersion, forming an all-in-focus image and estimating the depth information [199].

By elaborately tailoring the phase dispersion profiles of metalenses, researchers have developed a neural-network-based light-field imaging system capable of 4D image reconstruction (3D spatial information and additional spectral information), shown in Figure 8c [200]. Objects with slight spatial differences or spectral differences can be distinguished by rendering sub-images with spectral super-resolution algorithms. Figure 8d demonstrates a depth-sensing and imaging system that integrates light-field imaging and active structured light by using a broadband achromatic metalens array [201]. The proposed computational imaging approach-based meta-optics significantly reduces the total length of the imaging system, although it comes at the expense of resolution and with computational costs. Meta-optical applications in computational imaging take an essential step towards ultra-small on-chip optical systems, which may produce novel applications in endoscopy and brain imaging. In terms of the practical applications, smart vision (such as that of AR/VR devices) represented by computational metasurfaces is developing rapidly. The progress of some key technologies such as metasurface-based multicolor holograms [202], image classification [203], eye-tracking, and multi-functional display [204] have also proven their great potential, reliability, and transformative characteristics. Undoubtedly, meta-optical devices driven by smart vision have a huge future market. However, the process from research to market may be complex and difficult [205]. It can be expected that in the future, high-performance, high-efficiency, and multi-functional integrated meta-devices will be the key to the transition from theoretical research to practical applications centered on meta-optics and new optical physics.

### 4.2. Image Processing

**Figure 9:**
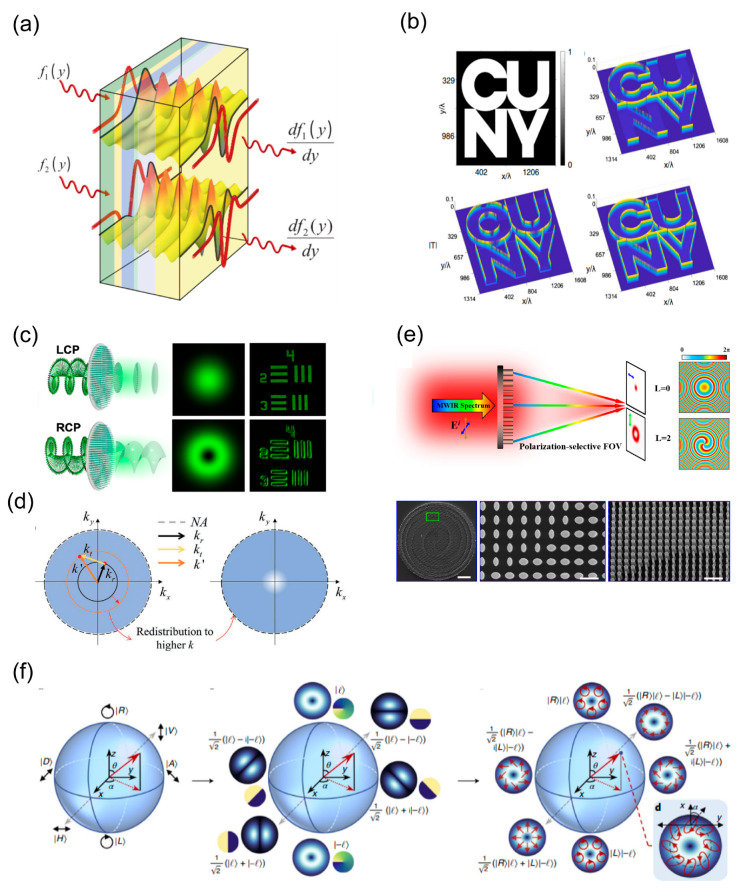
Metasurface-based images processing using structured light field. (**a**) Computational metamaterial devices based on the Green’s function method [209]. (**b**) Polarization-dependent edge-enhanced imaging [149]. (**c**) Schematic of the Fourier transform setup for spatial filtering (upper panel), and results of conversion between bright-field and phase-contrast imaging modes [210]. (**d**) Edge-enhancement effect of the spiral metalens [148]. (**e**) Schematic of the polarization-controlled broadband achromatic spiral metalens [157]. (**f**) Geometric representation of paraxial structured light [212].

Fast and reliable large-scale image processing is highly desirable due to its important applications in various domains such as object identification, machine vision, and artificial intelligence [206]. Meta-optics has offered a promising pathway to achieve compact and integrated photonic devices to address the limitations of conventional image processing [207]. Metasurface-enabled computing trends have had significant applications in optical differential operations and edge-enhanced imaging [208]. Since there is no analog-to-digital conversion or any other system delays, when an electromagnetic field passes through designed metamaterials with computing functions, the results of mathematical operations can be quickly obtained. Figure 9a presents the metamaterial-based computing configurations for mathematical operations (such as spatial differentiation, integration, or convolution) [44]. Mathematical operations (such as differential operations) can be performed in the Fourier domain using computational metasurfaces via green function methods. Another approach is the use of the spatial impulse response of the optical signal output through the multi-layer film to synthesize the mathematical operations that need to be performed. In terms of image processing, bright-field imaging and phase-contrast imaging are the two most representative working modes in optical imaging systems which can extract different kinds of morphological information about objects [209]. In particular, edge-state detection on images can obtain information that human eyes cannot perceive due to the presence of strong environmental and background noise. At the same time, computing power can be saved in the image processing system, and in-depth information analysis can be performed efficiently. Figure 9b demonstrates polarization-dependent edge-enhanced imaging performedby modulating the amplitudes of different polarized fields using asymmetric metasurface [149]. The designed non-local metasurfaces have successfully performed first-order and second-order derivative operations. These efforts in computational meta-optics demonstrate the feasibility of similar mathematical operations and provide ideas for efficient and fast image processing. Additionally, multi-functional integration of devices has always been in hot demand for parallel imaging processing, using a single image processing system to simultaneously achieve bright field and edge-state-enhanced imaging. As shown in Figure 9c, by introducing a spin-controlled bi-functional metasurface to a conventional 4*f* Fourier filtering system, imaging systems can perform isotropic edge-enhanced imaging and bright imaging based on the polarization state of the incident light, respectively [210].

Optical filter image processing based on the 4*f* system is not conducive to miniaturization, integration and portability. Therefore, single-layer metasurfaces that simultaneously perform derivative operations and imaging are highly desired to overcome that limitation. By introducing the desired phase profiles into the metalenses, the PSF can be flexibly modulated, resulting in novel image processing. Figure 9d demonstrates the schematic principle of a vortex-focused metalens performing spatial differentiation and edge-enhanced imaging, which originates from the light energy redistributions in the Fourier domain [148]. Edge-enhanced imaging operating at multiple discrete wavelengths in the visible range has been demonstrated; however, it inevitably suffers from chromatic aberration due to failure at wavelength dispersion control. To further promote the universality of the application of computational meta-optics, forward-looking research is needed to realize broadband achromatic edge-enhanced imaging and mathematical operations (such as differentials, integrals, etc.). As shown in Figure 9e, researchers proposed a polarization-controlled broadband achromatic vortex-focused metalens which can implement full-color edge-enhanced imaging and bright imaging with high efficiency [158]. In addition, the LC-based electric-driven polarization meta-optics paradigm has been demonstrated for tunable edge-enhanced images [211]. In a strict sense, all light beams have structure jointly defined by their polarization pattern, phase profile, and intensity distribution. The efforts in controlling and generating structured light have fueled its fundamental advances and applications alike. Figure 9f offers the fundamental representations of structured light fields. Analogous to the Poincare sphere of polarized light, the higher-order Poincare sphere is used to describe all vector modes described by the tensor product of a particular combination of the polarization and OAM states [212]. Recent advances have demonstrated that metasurfaces are excellent platforms for the generation and manipulation of structured light fields. Multi-functional metalenses capable of manipulating structured light beams may have novel and disruptive applications in computational imaging and parallel-image progress. More significantly, parallel to meta-optical applications is the deeper understanding of the possibilities that structured light brings.

## 5. Conclusions

Meta-optics has paved the promising way for ultimate miniaturization and multifunctionalities of optical components due to the novel concept and fundamentals beyond conventional optics. Metasurfaces have been manifested as excellent platforms for developing revolutionary optical elements owing to their powerful capacity to shape light beams with desired functionalities, ranging from initial wavefront control to optical analog computing. Indeed, meta-devices and their applications in the parallel control of light beams and ultralight optics have demonstrated disruptive characteristics due to the control over phase, amplitude, and polarization [158,165,170]. Here, we have reviewed recent achievements in meta-optical physics, meta-devices and their applications in computational imaging and image processing. We elaborate the fundamentals and principle of light manipulation, typical meta-devices (metalenses and vortex metalenses) and their key applications.

However, there are still challenges to the possible future development direction of the proposed metalenses. In some integrated designs, precise alignment presents process and operational challenges. Broadband achromatic focusing of single-layer metalenses has proven to be promising for imaging applications, but the achievable achromatic bandwidth is limited by some fundamental boundaries and fabrication challenges. As the sizes, bandwidth, and NAs of a metalens increase, larger group delays are required, resulting in the requirement of high aspect ratios for the fabricated meta-atoms. At the same time, from a manufacturing point of view, using meta-atoms of a greater height is an effective way to improve the work bandwidth of metalenses. However, the recent fabrications with high aspect ratios (such as 100:1) remain [183]. Furthermore, the high degree of freedom has further enhanced meta-devices’ multi-functionalities, work efficiencies and bandwidth, albeit at the expense of computational memory and durations. Simulation time increases dramatically as design degrees of freedom increase. Last but not least, the integrated packaging of on-chip meta-devices is very important, but the packaging materials around the patterns may cause a reduction in the devices’ efficiencies.

With the development of structured light modulation and the breakthrough of nano-fabrication, the meta-optical focus has gradually shifted to a focus on inter-disciplinary frontier science. The advances in the application of optical metasurfaces in quantum light generation and detection are very inspiring. For instance, Lee C. Bassett et al. propose an immersion metalens integrated on a diamond substrate instead of the conventional high-NA objective lens which collimates the emission of solid-state quantum light source in the substrate and improves the efficiency of photonic collection. The immersion metalens has demonstrated the potential of controlling light–matter interactions for quantum emitters [213]. Recently, researchers have proposed a graphene-based plasmonic metasurface to achieve electric-driven dynamic control of complex amplitudes [214]. Patrice Genevet et al. have proposed the concept of a conformal metasurface, resulting in meta-optics being applied to arbitrary geometrical boundaries [215]. This work offers a novel idea for the fusion of meta-optics and conventional refractive optics. The continuous innovation of novel concepts, optical physics, and key applications make meta-optics still vivid and prosperous. Underpinned by advances in meta-optical physics and the structured light field, we envision that the main efforts in meta-optics will focus on exploring the limits of light field control, integrating multi-functional computational imaging and parallel image progressing in on-chip systems, and improving the working efficiency and bandwidth of optical meta-devices about five years from now.

## Data Availability

Not applicable.
